# Clinical Outcomes of Hearing Aid Use in Moderate to Severe Sensorineural Hearing Loss: A Cross-Sectional Study from Romania

**DOI:** 10.3390/healthcare14010112

**Published:** 2026-01-02

**Authors:** Liviu Lucian Padurean, Horatiu Eugen Ștefanescu, Calin Muntean, Vasile Gaborean, Ioana Delia Horhat

**Affiliations:** 1 Doctoral School, “Victor Babeș” University of Medicine and Pharmacy, Eftimie Murgu Square 2, 300041 Timișoara, Romania; lucian.padurean@umft.ro; 2 Municipal Clinical Hospital, Pricazului Str. 16, 335700 Orăștie, Romania; 3 Discipline of Otorhinolaryngology, Department IX, “Victor Babeș” University of Medicine and Pharmacy, Eftimie Murgu Square 2, 300041 Timișoara, Romania; deliahorhat@yahoo.com; 4 Medical Informatics and Biostatistics, Departament III-Functional Sciences, “Victor Babeș” University of Medicine and Pharmacy, Eftimie Murgu Square 2, 300041 Timișoara, Romania; 5 Thoracic Surgery Research Center, “Victor Babeş” University of Medicine and Pharmacy Timişoara, Eftimie Murgu Square No. 2, 300041 Timişoara, Romania; vasile.gaborean@umft.ro; 6 Department of Surgical Semiology, Faculty of Medicine, “Victor Babeş” University of Medicine and Pharmacy Timişoara, Eftimie Murgu Square No. 2, 300041 Timişoara, Romania

**Keywords:** hearing aids, WRS, IOI-HA, HHIA, THI, tone audiometry and rehabilitation

## Abstract

**Background/Objectives**: This study aims to explore both the psychosocial outcomes of hearing aid use and the factors that differentiate users from non-users among older adults with sensorineural hearing loss (SNHL) in Romania. **Methods**: We conducted a cross-sectional, comparative study with follow-up, including 201 patients aged between 49 and 92 years (mean age 70.76 ± 11.86 years), diagnosed with moderate to severe SNHL, evaluated between 1 November 2023, and 30 November 2024, at the Municipal Clinical Hospital Orăștie, Romania. Audiological assessment involved pure-tone audiometry and speech testing. Outcome measures included the Word Recognition Score (WRS), International Outcome Inventory for Hearing Aids (IOI-HA), Hearing Handicap Inventory for Adults (HHIA), Tinnitus Handicap Inventory (THI), and the Self-Esteem Scale (SES). **Results**: Of the 201 patients, 105 (52.2%) accepted hearing aid (HA) fitting and 96 (47.8%) declined. No significant differences were found in age (*p* = 0.565) or sex (*p* = 0.476) between groups. HA users reported significantly lower perceived handicap (HHIA: 46.48 ± 24.83 vs. 77.74 ± 28.02, *p* = 0.015) and higher self-esteem scores (SES: 35.68 ± 4.88 vs. 23.03 ± 4.90, *p* < 0.001), while tinnitus-related distress (THI) did not differ significantly (*p* = 0.785). Word recognition scores improved significantly post-fitting across all degrees of hearing loss: moderate (48.52% → 86.13%), moderately severe (47.47% → 85.31%), and severe (47.55% → 85.46%), all *p* < 0.001. **Conclusions**: Hearing aid use in older adults with SNHL was associated with significant improvements in speech perception and reduced perceived hearing handicap. These benefits were consistent across all severity levels and were independent of unilateral or bilateral device use. The difference in self-esteem observed between users and non-users may reflect pre-existing psychological factors influencing HA adoption, underlining the importance of personalized counseling in hearing rehabilitation.

## 1. Introduction

Hearing loss is a highly prevalent disease among older people worldwide, with almost 5% of the global population being affected by it. It has been reported that 466 million people are affected worldwide (according to World Health Organization data), a number expected to rise to 630 million by 2030 [1,2]. Age-related hearing loss, or presbycusis, is characterized by progressive, symmetrical sensorineural hearing decline, often beginning in the fourth decade of life and becoming clinically significant after the age of 65 [3,4].

Hearing aids (HAs) remain the primary non-invasive intervention for adults with moderate to severe sensorineural hearing loss (SNHL), with consistent evidence supporting their role in improving speech perception, tinnitus distress, and psychosocial well-being. Clinical guidelines emphasize that individualized hearing aid fitting and rehabilitation lead to measurable improvements in communication and quality of life, especially when guided by best-practice protocols and outcome monitoring tools [5,6].

Multiple studies have demonstrated that HA use can alleviate both emotional and social distress associated with hearing impairment, including symptoms such as depression, loneliness, and dissatisfaction with perceived health status [7,8,9]. Furthermore, tinnitus severity has been shown to improve following auditory stimulation through HA use, particularly in individuals with concurrent hearing loss, regardless of whether the fitting is unilateral or bilateral [10,11]. Despite the proven benefits of HAs in enhancing communication and overall quality of life in those with sensorineural hearing loss, adoption rates remain low. Aside from barriers such as cost and limited access to audiological care, many individuals avoid using hearing aids due to stigma, skepticism about their effectiveness, or dissatisfaction with their performance—especially in challenging listening environments such as background noise [12,13].

In Romania, recent data suggest a rising prevalence of SNHL in the older population, increasing from 1.43% in 2018 to a projected 2.56% by 2050 [14]. While the benefits of hearing aids are well established, there is growing interest in understanding why many individuals still choose not to use them. This study focuses not only on the psychosocial impact of hearing aid use but also on the clinical and individual factors that distinguish users from non-users, within the specific context of the Romanian healthcare system. The uptake of HA among older patients remains limited and is being influenced not only by clinical factors but also by socioeconomic barriers in our country. Financial constraints play a central role, especially in rural and semi-urban areas where access to subsidized hearing devices and specialized audiological services is minimal. Low public awareness and the social stigma associated with hearing aid use further hinder acceptance, as previously reported in national and international studies [2,14]. In countries with advanced healthcare systems, such as Singapore, evidence showed that government-funding programs significantly increased both HA acquisition and compliance rates [15], suggesting that similar systemic support could have a positive impact in Romania as well. A recent study from Romania reported that elderly individuals living in care institutions are more likely to suffer from untreated hearing loss, often in the context of cognitive impairment [2,3,5]. However, the direction of this association remains uncertain, as cognitive decline may also reduce awareness of auditory difficulties. These findings emphasize how socioeconomic conditions and limited access to auditory care can negatively affect broader health outcomes [14].

### Study Objectives

This study addresses these gaps by evaluating the effectiveness of hearing aids in older Romanian patients diagnosed with moderate to severe SNHL. Unlike many previous investigations, that rely exclusively on objective audiometric measures, our study presents a comprehensive analysis of both objective tests (Word Recognition Score (WRS)) and [16] as well as subjective scales, including the Hearing Handicap Inventory for Adults (HHIA) [17], the Tinnitus Handicap Inventory (THI) [18], the International Outcome Inventory for Hearing Aids (IOI-HA) [19], and the Self-Esteem Scale (SES) [20]. These tools have been previously validated and used internationally [5,21,22,23,24,25], providing a multidimensional view of auditory function and quality of life.

One of the most important aspects of this study lies in the comparison between patients who accepted HA fitting and those who declined it, offering valuable insights into potential predictors of adherence and satisfaction. Moreover, this study presents outcomes depending on the severity of hearing loss, contributing to a more individualized understanding of intervention effects. This approach aligns with recent calls in the literature to move beyond group averages and focus on personalized auditory rehabilitation strategies [24,26,27].

Ultimately, the aim of this research is to strengthen the evidence base for HA adoption and effectiveness in the Romanian healthcare system and offers potential guidance for clinicians. At the same time, it highlights the crucial need to incorporate both audiological and psychosocial aspects into hearing rehabilitation—a holistic approach increasingly recognized as vital for enhancing outcomes in older patients with sensorineural hearing loss [28,29].

## 2. Materials and Methods

A total of 201 patients diagnosed with sensorineural hearing loss (SNHL) were included in the study, conducted at the Clinical Hospital of Orăștie, Romania, between 1 November 2023, and 30 November 2024. Participants were consecutively recruited from patients attending the Otorhinolaryngology Department of the Municipal Clinical Hospital of Orăștie, primarily for evaluation of hearing loss or related auditory complaints (e.g., communication difficulties, tinnitus). None were hospitalized for unrelated medical reasons. This recruitment approach ensured that all participants were evaluated within an audiological context, minimizing selection bias from unrelated hospital admissions. Participants were initially classified into two groups based on hearing aid (HA) use: users and non-users. The study followed a cross-sectional, comparative design, with a follow-up assessment conducted 30 days after the initial evaluation. Within each group, patients were further stratified by hearing loss severity into three categories: moderate, moderately severe, and severe. Eligibility criteria required participants to be native Romanian speakers. Exclusion criteria included:

- abnormal otoscopy,
- an air–bone gap greater than 5 dB at 500, 1000, and 2000 Hz,
- and technical defects of the hearing aids.
- only patients presenting with either unilateral hearing loss or bilateral hearing loss with symmetrical severity (i.e., the same degree of hearing impairment in both ears) were included.

None of the participants in the non-user group had any prior experience with hearing aids. All individuals were first-time candidates for amplification and were introduced to the possibility of hearing aid fitting during their evaluation at the Otorhinolaryngology Department.

Not all patients presented with bilateral SNHL. The study cohort included both unilateral and bilateral SNHL cases. Similarly, among HA users, some were fitted unilaterally and others bilateral.

To minimize the risk of bias, only patients with bilateral hearing loss who presented with symmetrical degrees of hearing impairment and who used bilateral hearing aids were included in the analysis.

The patients’ age and gender were recorded, and they were evaluated using several standardized measures, including Word Recognition Score (WRS) [16], the International Outcome Inventory for Hearing Aids (IOI-HA) [19], the Hearing Handicap Inventory for Adults (HHIA) [17], the Tinnitus Handicap Inventory (THI) [18] and The Self-Esteem Scale (SES) [20]. All questionnaires have been translated into Romanian. Patients underwent their initial evaluation at the first clinical encounter.

Patients who declined HA fitting also refused to undergo initial WRS testing. Consequently, WRS assessments were performed only in the group that accepted HA fitting, both prior to device placement and at 30-day follow-up.

At this second time point, the IOI-HA were recorded as well. In order to facilitate interpretation of the results, it is important to note that higher scores on the HHIA and THI scales reflect a greater perceived handicap and tinnitus-related distress. In contrast, higher scores on the SES, IOI-HA, and WRS scales indicate better self-esteem, more favorable hearing aid outcomes, and improved speech recognition, respectively.

Audiological evaluations such as WRS testing, were conducted in a sound-treated room using a calibrated clinical audiometer. Word recognition testing was conducted using monosyllabic word lists presented at individually selected suprathreshold levels between 65 and 90 dB HL, in accordance with clinical protocols recommending adjustment based on each patient’s pure-tone thresholds to ensure optimal audibility [30]. The tests were carried out separately for each ear using standard supra-aural headphones. Although some technical parameters were recorded by the device (e.g., impedance, output calibration), these are not relevant for interpretation and were therefore not detailed here. The booth met ANSI standards for ambient noise control, and the procedure followed current clinical guidelines. The degree of hearing loss was classified based on the pure-tone average (PTA) calculated across 500, 1000, 2000, and 4000 Hz, using the WHO classification: moderate (41–60 dB HL), moderately severe (61–80 dB HL), and severe (>81 dB HL).

### 2.1. Audiometer Check

We used an audiometer (SMART 130 (Inmedico A/S, Lystrup, Danemarca)) that was calibrated between 15 May 2023, and 15 December 2024. The speech audiometer specifications were as follows: power output 16 W, signal-to-noise levels between 65 and 90 dB HL, speaker drivers consisting of a 0.5″ 8 Ω tweeter and a 3″ 4 Ω woofer, frequency response 80–20,000 Hz, and dimensions 113 × 207 × 120 mm. The measurements were conducted at 500, 1000, 2000, and 4000 Hz in a quiet environment. Testing was performed monaurally using headphones, starting at 45 dB and increasing in 5–15 dB increments until 100% speech intelligibility was achieved.

The study was conducted in accordance with the principles of the Declaration of Helsinki and was approved by the Ethics Committee of the “Victor Babeș” University of Medicine and Pharmacy Timișoara (Approval No. 70/1 November 2023).

### 2.2. Statistical Analysis

For the statistical analysis and interpretation of results, we utilized IBM SPSS Statistics 25 software for Windows (IBM, Armonk, NY, USA). Descriptive statistics were used to summarize demographic and clinical characteristics, with data expressed as mean ± standard deviation (M ± SD) for continuous variables and as frequencies and percentages for categorical variables, and Chi square tests where necessary. Comparisons between groups were performed using independent samples *t*-tests and one-way analysis of variance (ANOVA), followed by post hoc comparisons where appropriate. A *p*-value of less than 0.05 was considered statistically significant.

## 3. Results

### 3.1. Participant Characteristics

This study included 201 patients diagnosed with SNHL. Among them, 105 patients (52.2%) agreed to use a HA, while 96 patients (47.8%) declined. The overall mean age of the cohort was 70.76 ± 11.86 years. There were no significant differences in age between males and females (71.31 ± 11.19 vs. 70.35 ± 12.38, *p* = 0.564).

Of the 201 patients with hearing loss, 105 (52.2%) agreed to use a HA, while the remaining 96 (47.8%) declined. No significant differences were found in the mean age between the two groups (71.23 ± 12.29 years vs. 70.26 ± 11.43 years, *p* = 0.565), nor in the proportion of male patients (48.8% vs. 51.2%, *p* = 0.476).

The demographic characteristics of the three groups are presented in Table 1.

### 3.2. Audiological and Psychosocial Outcomes in HA Users vs. Non-Users

Patients who accepted HA had a mean age of 71.87 ± 10.87 years, while those who declined had a mean age of 70.06 ± 14.67 years, with no statistically significant difference between the two groups (*p* = 0.372). Patients who refused HA also declined WRS testing, choosing to complete only the study questionnaires.

From 201 patients, 84 (41.79%) were affected by tinnitus, 87 patients (43.28%) presented with a hearing handicap, and 94 patients (46.76%) had a lower or moderate self-esteem.

Three scores were compared between patients with hearing aids and those without at the time of the initial consultation. The results are summarized in Table 2.

Among the patients using HA, 69 (65.71%) had a unilateral device and 36 (34.28%) had a bilateral device. There were no significant differences in the mean age between the two groups (71.87 ± 10.87 vs. 70.06 ± 14.67, *p* = 0.518), nor in the proportion of male patients (43.5% vs. 33.3%, *p* = 0.402).

The degree of hearing loss was analyzed in patients with unilateral and bilateral HA. The results are presented in Table 3.

All patients with hearing loss who used HA were evaluated prior to treatment initiation using the WRS score, which showed no significant differences between unilateral and bilateral users (49.28% ± 30.94% vs. 47.08% ± 25.39%, *p* = 0.716). No significant differences were observed after fitting the HA either, as assessed by the WRS score (84.55% ± 22.99% vs. 89.17% ± 18.72%, *p* = 0.302).

The variation in HHIA, THI, and SES scores among patients with unilateral and bilateral HA is presented in Table 4.

The variation in IOI-HA scores after fitting unilateral or bilateral devices showed no significant differences between the two groups (30.12 ± 4.2 vs. 39.72 ± 5.24, *p* = 0.699).

Among the 105 patients with hearing loss, 61 (58.1%) had moderate hearing loss, 18 (17.1%) had moderately severe hearing loss, and 26 (24.8%) had severe hearing loss.

The variation in scores showing significant differences among the three degrees of hearing loss severity is presented in Table 5.

An individual analysis was performed comparing patients without HA to those with SNHL using HA. The results are presented in Table 6.

### 3.3. Evaluation of WRS

The WRS was applied only to patients who accepted HA. It was assessed at the initial consultation and subsequently after HA fitting.

At baseline, patients had a mean WRS of 48.52% ± 29.05%, which increased to 86.13% ± 21.65% post-fitting, showing a statistically significant difference between the two time points (*p* < 0.001).

The variation in WRS improvement by degree of hearing loss is presented in Table 7.

## 4. Discussion

### 4.1. Hearing Aid Acceptance and Barriers to Adoption

The observed HA adoption rate in our cohort aligns with international trends. Despite the demonstrated benefits of amplification, HA uptake remains suboptimal. In Europe, studies show that even in well-funded systems like Norway, nearly one-third of patients with disabling hearing loss did not use hearing aids, highlighting barriers unrelated to audiological severity [31].

Several studies have shown that financial constraints, stigma, and limited perceived benefit remain the predominant reasons for refusing hearing aids [32]. Psychological factors, including denial of hearing loss and negative attitudes toward amplification, have also been identified as key predictors of non-adoption [33]. Moreover, large-scale reviews indicate that perceived communication need, comfort, and self-efficacy play a more decisive role in hearing aid uptake than audiometric severity alone [34,35].

Our findings confirmed that HA users reported significantly lower handicap scores (HHIA) and higher SES compared to non-users. Similar results have been observed in previous studies, where amplification effectively reduced the psychosocial burden associated with hearing loss in older adults [17,21]. Moreover, research has shown that hearing aid use contributes to better emotional well-being and decreased social isolation, reinforcing its importance for maintaining quality of life in aging populations [1,29].

Although our SES results were higher among HA users, these findings should be interpreted with caution, as the assessment was performed prior to HA fitting. This suggests that self-esteem may act more as a facilitator of help-seeking behavior than as a direct consequence of amplification. Previous studies have demonstrated that individuals with higher self-esteem and stronger internal locus of control are more likely to pursue hearing rehabilitation and to report greater satisfaction with hearing aids [32,36]. Moreover, personality-related factors such as resilience and perceived control have been associated with proactive hearing health behaviors and better adjustment to hearing loss [37]. Together, these findings indicate that self-esteem may serve as a psychological predictor of HA adoption and adherence rather than an outcome of amplification alone.

### 4.2. Audiological Outcomes and Speech Perception

One of the most significant findings in our cohort was the improvement in WRS following HA fitting. Across all degrees of hearing loss (moderate, moderately severe, and severe), WRS increased from a mean baseline of 48.52% to 86.13% after 30 days of device use, with *p* < 0.001 in all subgroups. These results strongly support the role of HAs in enhancing speech perception. Similar findings have been reported by Luo et al., who demonstrated that bone-conduction hearing aids significantly improved speech recognition and quality of life in adults with single-sided deafness [6]. Likewise, Kim et al. showed that both traditional hearing aids and personal sound amplification devices enhanced word and sentence recognition in adults with unilateral hearing loss [30].

Our study also revealed that post-fitting WRS improvements were consistent across severity groups. This is an important observation, as it suggests that patients with even severe SNHL can benefit from amplification if fitted early and appropriately. Other studies noted that proper calibration and patient-specific fitting strategies lead to significant gains in speech intelligibility, even among those with mixed or more advanced hearing loss [5,9].

Notably, no significant differences were observed between unilateral and bilateral HA users in any major outcomes—WRS, HHIA, IOI-HA, or THI. While bilateral fittings are theoretically superior for sound localization and speech-in-noise perception, real-world benefits often depend on cost, motivation, and individual preference. Previous investigations have shown that perceived benefit, comfort, and usability frequently outweigh the acoustic advantages of bilateral amplification in determining long-term satisfaction and adherence [38,39]. Furthermore, research indicates that emotional well-being and subjective perception of communication benefit are stronger predictors of HA satisfaction than the number of devices used [40].

### 4.3. Psychosocial Dimensions of Hearing Loss and Amplification

Regarding tinnitus outcomes, no significant differences in THI scores were observed between HA users and non-users in our study. This finding aligns with previous research showing that while amplification can alleviate tinnitus perception in some individuals, outcomes remain variable and are often optimized when combined with counseling or sound therapy [5,13]. Moreover, improvements in tinnitus distress tend to be greater among users who receive additional psychological or cognitive-behavioral support alongside standard HA fitting [41].

An additional layer of interpretation stems from the observed progression of HHIA, THI, and SES scores relative to hearing loss severity. As hearing loss advanced from moderate to severe, psychosocial burden also increased, while self-esteem declined. Previous research has demonstrated that greater auditory thresholds are associated with lower social participation, higher emotional distress, and poorer overall well-being [1,42,43]. Furthermore, best-practice guidelines highlight that for patients with more profound hearing loss, audiological interventions alone are insufficient; comprehensive rehabilitation should include psychological counseling and communication training to achieve optimal functional recovery [5,29].

These observations align with the broader consensus that auditory rehabilitation should not rely solely on technical fitting but should integrate psychological support, patient education, and counseling to maximize rehabilitation outcomes. Evidence from multidisciplinary and patient-centered models has shown that combining amplification with structured counseling and self-management strategies leads to superior functional and emotional results in adults with hearing loss [29,44,45,46].

In summary, our findings align closely with the international literature emphasizing that hearing aid use significantly improves speech perception and reduces psychosocial distress, particularly in older adults with SNHL. However, the benefits are multifaceted and often depend on individual motivation, psychosocial context, and the presence of support systems. WRS gains were robust across severity groups, reinforcing the efficacy of amplification in restoring speech intelligibility. Still, the variable outcomes in tinnitus and SES measures highlight the need for ongoing psychological and rehabilitative support in comprehensive hearing care.

### 4.4. Study Limitations

Despite these results, our study has several important limitations. It was carried out in a single hospital, which may limit the applicability of the findings to broader populations. Although the sample size was moderate, it might not fully represent the range of sociodemographic factors. One limitation of this study is that follow-up data were only collected for patients who received hearing aids, while those who declined were evaluated at a single time point. As a result, comparisons between groups should be interpreted with caution due to differences in temporal data availability. The short 30-day follow-up represents a limitation of this study. Most patients did not return for later evaluations, reflecting the general challenges of maintaining long-term follow-up in the Romanian healthcare system due to limited health literacy and poor adherence to medical recommendations. It is important to emphasize that this study was conducted in the aftermath of the COVID-19 pandemic, which significantly affected healthcare systems in Romania and worldwide [47,48]. Evidence from the United States indicates that nearly 20% of older adults with hearing loss postponed audiology appointments during the pandemic, resulting in delays in diagnosis and treatment [49]. These observations align with our clinical experience, where many older patients deferred consultations due to infection concerns, leading to deteriorated hearing thresholds and diminished chances for effective rehabilitation.

## 5. Conclusions

This study demonstrates that hearing aid use in older Romanian adults with sensorineural hearing loss significantly improves speech perception and reduces perceived handicap, with consistent gains across all severity levels. While audiological benefits such as improved word recognition are clear, psychosocial outcomes like self-esteem and tinnitus relief are more variable, underscoring the need for personalized, integrative rehabilitation strategies that go beyond amplification alone. These findings emphasize the importance of early intervention, patient-centered care, and systemic support in optimizing hearing outcomes in aging populations.

## Figures and Tables

**Table 1:** Demographic aspects.

Score	Moderate n = 73	Moderately Severe n = 58	Severe n = 70	*p*
Age (M ± SD)	71.51 ± 12.06	72 ± 14.2	70.08 ± 11.81	0.851
Gender (Male)	22 (36.1%)	9 (50%)	11 (42.3%)	0.549

M = mean; SD = standard deviation.

**Table 2:** Comparative tests results non HA vs. HA.

Score	Without HA n = 96	With HA n = 105	*p*
HHIA (M ± SD)	77.74 ± 28.02	46.48 ± 24.83	0.015
THI (M ± SD)	40.58 ± 19.84	40.96 ± 20.45	0.785
SES (M ± SD)	23.03 ± 4.9	35.68 ± 4.88	<0.001

HA = hearing aid; M = mean; SD = standard deviation; HHIA = Hearing Handicap Inventory for Adults; THI = Tinnitus Handicap Inventory; SES = the Self-Esteem Scale.

**Table 3:** Unilateral vs. bilateral hearing loss in patients with HA.

Severity	Unilateral HA n = 69	Bilateral HA n = 36	*p*
Moderate	37 (53.6%)	24 (66.7%)	0.368
Moderately severe	14 (20.3%)	4 (11.1%)	
Severe	18 (26.1%)	8 (22.2%)	

HA = hearing aid; M = mean; SD = standard deviation.

**Table 4:** Mean scores in unilateral vs. bilateral HA.

Score	Unilateral HA n = 69	Bilateral HA n = 36	*p*
HHIA (M ± SD)	48.42 ± 25.91	43.06 ± 22.52	0.275
THI (M ± SD)	43.38 ± 20.7	36.33 ± 19.41	0.089
SES (M ± SD)	35.80 ± 4.7	35.44 ± 5.289	0.737

HA = hearing aid; M = mean; SD = standard deviation; HHIA = Hearing Handicap Inventory for Adults; THI = Tinnitus Handicap Inventory; SES = the Self-Esteem Scale.

**Table 5:** Comparison of audiological and psychosocial scores by hearing loss severity.

**Score (All Patients)**	**Moderate** **n = 73**	**Moderately Severe** **n = 58**	**Severe** **n = 70**	***p***
HHIA (M ± SD)	35.59 ± 17.05	49.44 ± 24.08	70.38 ± 24.13	<0.001
THI (M ± SD)	33.77 ± 14.67	42.94 ± 21.71	56.46 ± 22.87	<0.001
SES (M ± SD)	36.59 ± 3.06	36.44 ± 4.07	33 ± 7.45	0.005
**Score (only HA patients)**	**Moderate** **n = 12**	**Moderately severe** **n = 40**	**Severe** **n = 44**	***p***
IOI-HA (M ± SD)	30.82 ± 3.3	29.56 ± 5.6	28.31 ± 5.9	0.059

HA = hearing aid; M = mean; SD = standard deviation; IOI-HA = International Outcome Inventory for Hearing Aids; HHIA = Hearing Handicap Inventory for Adults; THI = Tinnitus Handicap Inventory; SES = the Self-Esteem Scale.

**Table 6:** Results in moderate hearing loss.

Scores	Moderate Hearing Loss Without HA, n = 12	Moderate Hearing Loss with HA, n = 61	*p*
HHIA (M ± SD)	32.67 ± 23.76	35.59 ± 17.05	0.614
THI (M ± SD)	27.83 ± 18.26	33.77 ± 14.67	0.223
SES (M ± SD)	27.25 ± 6.46	36.59 ± 3.06	<0.001
**Scores**	**Moderately severe hearing loss without HA, n = 40**	**Moderately severe hearing loss with HA, n = 18**	***p***
HHIA (M ± SD)	42.3 ± 22.91	49.44 ± 24.08	0.284
THI (M ± SD)	35.25 ± 16.75	42.94 ± 21.71	0.146
SES (M ± SD)	23.40 ± 4.46	36.44 ± 4.07	<0.001
**Scores**	**Severe hearing loss without HA, n = 44**	**Severe hearing loss with HA, n = 26**	***p***
HHIA (M ± SD)	74.25 ± 21.15	70.38 ± 24.13	0.486
THI (M ± SD)	48.91 ± 19.68	56.46 ± 22.87	0.149
SES (M ± SD)	21.55 ± 4.13	33.00 ± 7.45	<0.001

HA = hearing aid; M = mean; SD = standard deviation; IOI-HA = International Outcome Inventory for Hearing Aids; HHIA = Hearing Handicap Inventory for Adults; THI = Tinnitus Handicap Inventory; SES = the Self-Esteem Scale.

**Table 7:** Variation in WRS before and after HA.

Scores	WRS Before HA	WRS After HA	*p*
Moderate hearing loss, n = 61	57.78% ± 31.49%	90.23% ± 18.75%	<0.001
Moderately severe hearing loss, n = 18	49.10% ± 28.17%	80% ± 14.55%	<0.001
Severe hearing loss, n = 26	40.77% ± 28.41%	73.85% ± 27.43%	<0.001

## Data Availability

The original contributions presented in the study are included in the article, further inquiries can be directed to the corresponding author.
